# Alveolar Type II Epithelial Cell Dysfunction in Rat Experimental Hepatopulmonary Syndrome (HPS)

**DOI:** 10.1371/journal.pone.0113451

**Published:** 2014-11-24

**Authors:** Wenli Yang, Bingqian Hu, Wei Wu, Sachin Batra, Michael R. Blackburn, Joseph L. Alcorn, Michael B. Fallon, Junlan Zhang

**Affiliations:** 1 Division of Gastroenterology, Hepatology and Nutrition, Department of Internal Medicine, The University of Texas Health Science Center at Houston, Houston, TX, United States of America; 2 Department of Biochemistry and Molecular Biology, The University of Texas Health Science Center at Houston, Houston, TX, United States of America; 3 Division of Neonatology, Department of Pediatrics, The University of Texas Health Science Center at Houston, Houston, TX, United States of America; The Hospital for Sick Children and The University of Toronto, Canada

## Abstract

The hepatopulmonary syndrome (HPS) develops when pulmonary vasodilatation leads to abnormal gas exchange. However, in human HPS, restrictive ventilatory defects are also observed supporting that the alveolar epithelial compartment may also be affected. Alveolar type II epithelial cells (AT2) play a critical role in maintaining the alveolar compartment by producing four surfactant proteins (SPs, SP-A, SP-B, SP-C and SP-D) which also facilitate alveolar repair following injury. However, no studies have evaluated the alveolar epithelial compartment in experimental HPS. In this study, we evaluated the alveolar epithelial compartment and particularly AT2 cells in experimental HPS induced by common bile duct ligation (CBDL). We found a significant reduction in pulmonary SP production associated with increased apoptosis in AT2 cells after CBDL relative to controls. Lung morphology showed decreased mean alveolar chord length and lung volumes in CBDL animals that were not seen in control models supporting a selective reduction of alveolar airspace. Furthermore, we found that administration of TNF-α, the bile acid, chenodeoxycholic acid, and FXR nuclear receptor activation (GW4064) induced apoptosis and impaired SP-B and SP-C production in alveolar epithelial cells *in vitro*. These results imply that AT2 cell dysfunction occurs in experimental HPS and is associated with alterations in the alveolar epithelial compartment. Our findings support a novel contributing mechanism in experimental HPS that may be relevant to humans and a potential therapeutic target.

## Introduction

Pulmonary dysfunction is common in patients with chronic liver disease and may worsen outcomes [1]–[4]. The hepatopulmonary syndrome (HPS) is recognized as one important cause for abnormal oxygenation in cirrhosis that occurs in up to 32% of patients and significantly impairs quality of life and increases mortality [5]–[9]. The development of abnormal gas exchange in HPS is believed to be driven by vasodilatation and/or angiogenesis in the alveolar microcirculation which leads to mismatching of blood flow and ventilation [10], [11]. However, abnormal pulmonary function tests, including decreased total lung and diffusion capacity and ventilatory restriction have been observed in patients with advanced liver disease, particularly in HPS [3], [5], [12]. These findings suggest that the alveolar epithelial compartment may also be affected in patients with HPS and could contribute to gas exchange abnormalities.

The alveolar compartment is comprised of type I and type II alveolar epithelial cells (AT1 and AT2) that cover about 96% and 4% respectively of the internal air surface [13], [14]. AT1 cells, are large squamous cells with thin cytoplasmic extensions (50–100 nm) which form the air-blood barrier for gas exchange [14]. AT2 cells are smaller cuboidal cells, that play a critical role in the maintenance of lung function through producing surfactants and facilitating alveolar repair in response to injury [15]. Four surfactant associated proteins (SPs), SP-A, SP-B, SP-C and SP-D, are produced by AT2 cells [16]. Among them, pulmonary SP-C expression is restricted to AT2 cells, while SP-A, SP-B and SP-D are also produced outside the lung [17], [18]. Impaired AT2 cell integrity and surfactant protein production contributes to the development of a variety of lung diseases including COPD, ARDS and pulmonary fibrosis [19]–[21]. To date, no studies have evaluated the alveolar epithelial compartment in HPS.

Experimental biliary cirrhosis induced by common bile duct ligation (CBDL) in the rat reproduces the pulmonary vascular and gas exchange abnormalities of human HPS and has been extensively used as an animal model [8], [22]. CBDL is unique in causing HPS relative to models of prehepatic portal hypertension (partial portal vein ligation, PVL) [23], [24]. In CBDL animals, biliary obstruction triggers a systemic inflammatory response that results in elevated hepatic and serum levels of bile acids and increases in circulating levels of endotoxin and TNF-α [23]. Bile acids contribute to the development of liver injury by causing hepatocyte and biliary epithelial cell inflammatory responses and/or apoptosis [25]–[29]. Both bile acids and TNF-α have also been shown to reduce surfactant protein production and enhance apoptosis in AT2 cells [30]–[33].

In the present study, we evaluated alterations in the alveolar epithelial compartment, particularly AT2 cells, in experimental HPS relative to PVL and assessed pulmonary function test results from cirrhotic patients with or without HPS. In addition, we evaluated the effects of candidate mediators on AT2 cell functional properties *in vitro*.

## Materials and Methods

### Animals

Male Sprague-Dawley rats (200–250 g; Charles River, Wilmington, MA) were used in all experiments. The animals were anesthetized with intraperitoneal injection of xylazine and ketamine. CBDL and PVL were performed as previously described [10], [22]. Control animals underwent mobilization of the common bile duct or portal vein without ligation. The study was approved by The University of Texas at Houston Health Science Center Animal Welfare Committee and conformed to National Institutes of Health guidelines on the use of laboratory animals (PHS Assurance Number: A3413-01). All efforts were made to minimize suffering.

### Lung Fixation, Sampling and Processing

The animals were euthanized under anesthesia. The trachea was cannulated with a 15-gauge catheter and tied securely. The chest was opened, and right ventrical perfusion was accomplished with PBS to clear the pulmonary intravascular space. The left lung was removed and snap-frozen in liquid nitrogen. The right lung was inflated with 4% paraformaldehyde at a pressure of 20 cm H_2_O for about 5 minutes; the tracheal cannula was then closed to maintain airway pressure. The lung was fixed for 24 hours, paraffin- embedded and cut at a thickness of 5 µm for histology analysis. H&E staining was performed to evaluate the pulmonary morphological changes and measure alveolar mean chord length.

### Immunofluorescence, AT2 Cell Count and TUNEL Staining

Immunofluorescence (IF) staining of SP-C (Santa Cruz Biotechnology, cat# sc-13979 R in 1∶100 dilution) was performed as previously described [11]. Fluorescein secondary antibody and mounting medium with DAPI were from Vector Laboratories (cat# FI-1200 in 1∶200 dilution). The numbers of SP-C positive AT2 cells were counted in a blinded fashion under an immunofluorescence microscopy. Six animals were analyzed per group and ten to fifteen fields were counted per tissue section. For double-immunostaining, end-labeling of exposed 3′-OH ends of DNA fragments in paraffin-embedded tissue was undertaken with the TUNEL in situ cell death detection kit, Fluorescein (Roche Applied Science, Indianapolis, IN) according to manufacturer's instructions, which was followed by incubation with primary SP-C antibody (Santa Cruz Biotechnology, cat# sc-13979 R in 1∶100 dilution) for overnight at 4°C and then a Texas red secondary antibody (Vector Laboratories, cat# TI-1000 in 1∶200 dilution) in PBS for 0.5 hour at room temperature. Slides were mounted with Vectashield with DAPI.

### Lung Volume and Morphometric Assessments

The volumes of fixed right lung were measured with saline displacement method [34]. Morphometric changes were assessed by measuring mean chord length (Lm) on H&E stained lung sections with a computer-assisted image analysis system as previously reported [35]–[37]. Briefly, a set of test lines spanning across the alveolar space between alveolar septa were randomly placed on digital microscopic images of H&E lung sections. Lm was calculated automatically as the mean length of these chord line segments. Six animals were used in each group. Ten to fifteen fields were analyzed each section. The assessment was performed in a blinded manner.

### Preparation of Broncho Alveolar Lavage Fluids (BALFs)

The lung was sequentially lavaged three times through a tracheal catheter with PBS (8 ml/each time). The lavage fluids were pooled together and centrifuged at 300 g to remove cell contamination. The supernatants were collected, aliquoted and stored at −80°C for further analysis.

### AT2 Cell Isolation

Lung AT2 cells were isolated from sham and 3 wk CBDL rats using the modified methods as previously described [38]. After perfused through right ventricle with PBS, the lung was lavaged with PBS through the tracheal catheter, and digested with an enzyme solution containing 0.008% elastase (Worthington Biochemical Corp., Lakewood, NJ), 0.2% collagenase (Worthington Biochemical Corp), and 0.005% DNAse Type I at 37°C for 30 minutes. The lungs were then transferred to 4°C, and the enzyme solution was replaced with another PBS-enzyme solution containing 0.08% trypsin inhibitor (Sigma-Aldrich), 0.01% DNAse Type 1, 4% fetal bovine serum (Sigma-Aldrich). The lung lobes were then finely chopped and incubated in solutions, shaken on a rotary shaker for 25 minutes at 4°C. The mixture was then sequentially filtered through 100-, 40- and 15-mm nylon meshes and incubated in rat IgG-coated plastic Petri dishes for 60 minutes. AT2 cells were obtained by collecting unattached cells. The cell purity was determined by the SP-C IF staining of cell smear. Whole cell lysates were analyzed by western blotting using primary antibodies to cleaved caspase-3 (Asp175; Cell Signaling, cat# 9661 in 1∶1000 dilution) to evaluate AT2 cell apoptosis.

### Determination of TNF-α and Bile Acids levels

The concentrations of bile acid and TNF-α in plasma and BAL fluids were measured using a colorimetric assay kit (BQ Kits, San Diego, CA) and a ELISA kit (R&D Systems, Minneapolis, MN) following manufacture instructions.

### Cell Culture and Treatment

Murine lung epithelial cell lines MLE-12 (ATCC, Manassas, VA) were maintained and sub-cultured in DMEM- F-12 Medium with 10% FBS at 37°C in presence of humidified 95% air and 5% CO_2_ [39]. After starvation with 0.1% fetal bovine serum for 4 hours, cells were stimulated with recombinant rat TNF-a (R&D Systems), CDCA (Sigma, St. Louis, MO) and GW4064 (FXR/bile acid nuclear receptor agonist, Sigma) for 18–24 hours. Whole cell lysates were collected for total RNA or protein extraction.

### Western Blot Analysis

Western blot analysis of lung tissues or cell lysates were performed using primary antibodies for SP-A, B, C and D (Santa Cruz Biotechnology, cat# sc-13977; sc-13978; sc-13979R; sc-13980 in 1∶500 dilution), AQP5 (Millipore, cat# AB15858 in 1∶500 dilution), cleaved caspase-3 (Asp175; Cell Signaling, cat# 9661 in 1∶1000 dilution) and GAPDH (Cell Signaling, cat# 2118 in 1∶1000 dilution). Goat anti-rabbit horseradish peroxidase conjugated secondary antibody (Calbiochem, 1∶2000 dilution) and enhanced chemiluminescence substrate Pico-West luminol reagent (Thermo Scientific Pierce) were used to detect protein bands. GAPDH was included in all blots as a loading control. The density of autoradiographic signals of each band was assessed with a ScanMaker i900 scanner (Microtek Lab, Carson, CA) and quantitated with NIH Image J software. SP protein levels were normalized to GAPDH. The final quantification of SP proteins was summarized from at least three independent experiments.

### RNA Extraction and Quantitative Real-Time RT-PCR

Total RNA from lungs or MLE-12 cells were extracted with Trizol reagent (Invitrogen, Carlsbad, CA) according to the manufacturer's instructions and treated with RNase-free DNase I (Invitrogen) following the manufacturer's protocol. cDNA was prepared using the High Capacity cDNA Reverse Transcription kit (Life Technologies, Grand Island, NY). qRT-PCR analysis was performed using TaqMan Gene Expression Assays for SP- B and SP-C (Life Technologies, Grand Island, NY). Expression levels were normalized to the expression of 18S rRNA.

### Pulmonary Spirometry Tests in Liver Patients

Forced vital capacity (FVC), forced expiratory volume in 1 s (FEV1), and FEV1/FVC ratio were measured by spirometry in liver patients with or without HPS. Institutional Review Board Approval was obtained from the University of Texas- Houston.

### Statistical Analysis

Measurements are expressed as means ± SEM. Data were analyzed with the two-tailed Student *t* test or analysis of variance with Bonferroni correction for multiple comparisons between groups. Statistical significance was designated as *P*<0.05.

## Results

### The Effects of CBDL and PVL on Pulmonary Surfactant Protein Levels

**Figure 1:**
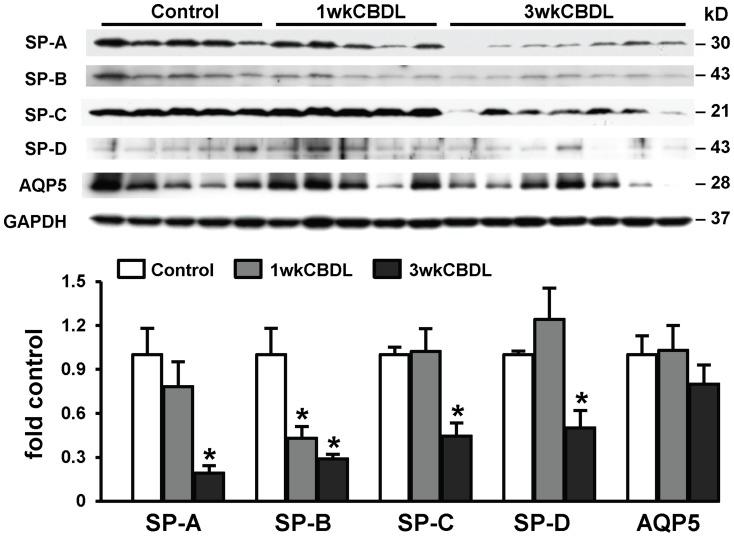
Pulmonary surfactant associated protein (SP) levels after CBDL. Lung tissues were obtained from common bile duct ligation (CBDL) animals at 1 and 3 weeks after ligation. The protein levels of SPs and aquaporin 5 (AQP5, an alveolar type I epithelial cell marker) in lung homogenates were assessed by western blots. Three independent experiments were performed. Top panel, representative immunoblots of SP-A, SP-B (precursor), SP-C (precursor), SP-D, AQP5 and GAPDH in animal lung. Bottom panel, the graphical summaries of protein levels for SP-A, SP-B, SP-C, SP-D and AQP5. SPs protein levels were normalized to the levels of GAPDH. SPs production was significantly decreased between 1 and 3 weeks after CBDL, whereas AQP5 protein levels remained unchanged. Values are expressed as means ± SME. * *P*<0.05 compared with control.

**Figure 2:**
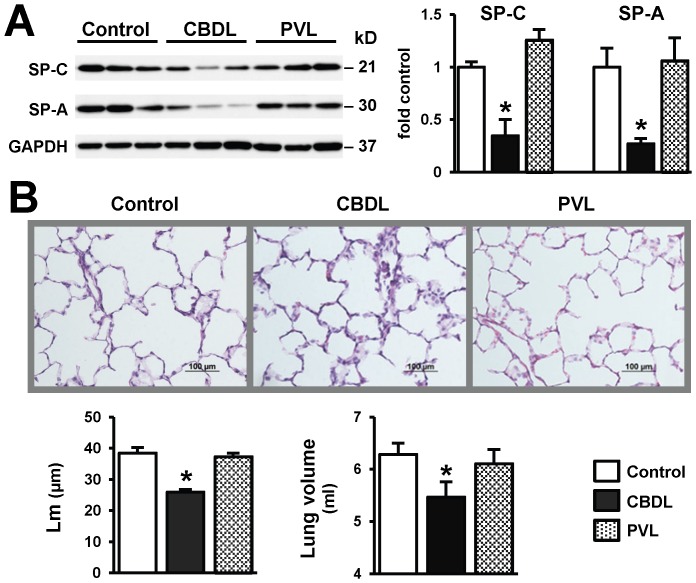
Comparison of pulmonary associated protein (SP) levels and morphologic changes in CBDL and PVL animals. (**A**) Representative immunoblots and graphical summaries of protein levels of SP-A, SP-C (precursor) and GAPDH in lung from 3-week CBDL and 3-week PVL animals. Three independent experiments were performed. SPs protein levels were normalized to the levels of GAPDH. Significant decreases in lung SP-A and SP-C levels were only seen after CBDL, not PVL. (**B**) Representative H&E images of animal lung and quantitation of alveolar mean cord length (Lm) and right lung volumes by saline displacement method. Six animals were used in each group. Ten to fifteen fields were counted per tissue section. The analysis was performed in a blinded manner. Relative to control or PVL, CBDL resulted in shrunk alveoli. There was a significant reduction in Lm and lung volumes in 3-week CBDL animal, which was not seen in control or 3-week PVL. Scale bar = 100 µm. Values are expressed as means ± SME. * *P*<0.05 compared with control.

To determine if lung production of surfactant proteins is affected by CBDL, we measured the protein levels of SP-A, SP-B and SP-C (pro-SP-C) in 1 and 3 wk CBDL animals (Figure 1). There was a significant reduction in pulmonary AT2 cell surfactant protein levels relative to sham operated animals, which developed between 1 and 3 weeks after CBDL as HPS developed (at 3 wk: SP-A 0.19±0.05 fold-control; SP-B 0.29±0.03 fold-control; pro-SP-C 0.45±0.09 fold-control, SP-D 0.50±0.12 fold-control, all p<0.05 *vs* sham) indicating reduced lung surfactant protein production. To evaluate whether similar events occur in the lung after PVL, we compared two representative surfactant proteins, SP-A and pro-SP-C protein levels in 3 wk CBDL and 3 wk PVL animals (Figure 2A). In contrast to 3 wk CBDL lungs where surfactant protein levels were decreased, the expression of SP-A and pro-SP-C levels remained unchanged in 3 wk PVL animals. To define if changes in AT2 cell surfactant production are part of a generalized effect, we measured lung AQP5 (a specific AT1 cell marker) levels. We observed no significant alteration in AQP5 protein levels after CBDL (Figure 1), supporting a unique effect on AT2 cells.

### Pulmonary Morphologic Changes after CBDL and PVL

To assess whether the reduction in surfactant proteins after CBDL is associated with morphologic alterations in the lung, we measured lung volumes using a water displacement method and quantified the average alveolar size expressed by mean chord length (Lm) in control and CBDL animals (Figure 2B). Relative to sham and PVL animals, total right lung volume (0.87 fold vs control, p<0.05) and mean chord length (25.9±0.9 vs 38.4±1.8 µm, p<0.05) decreased significantly after CBDL indicating a selective reduction of alveolar airspace. These findings show that reduced pulmonary surfactant protein levels after CBDL are accompanied by a defect in maintenance of alveolar integrity resulting in alveolar collapse and a reduction in lung volume.

### Pulmonary SP-C Expression and Localization after CBDL

**Figure 3:**
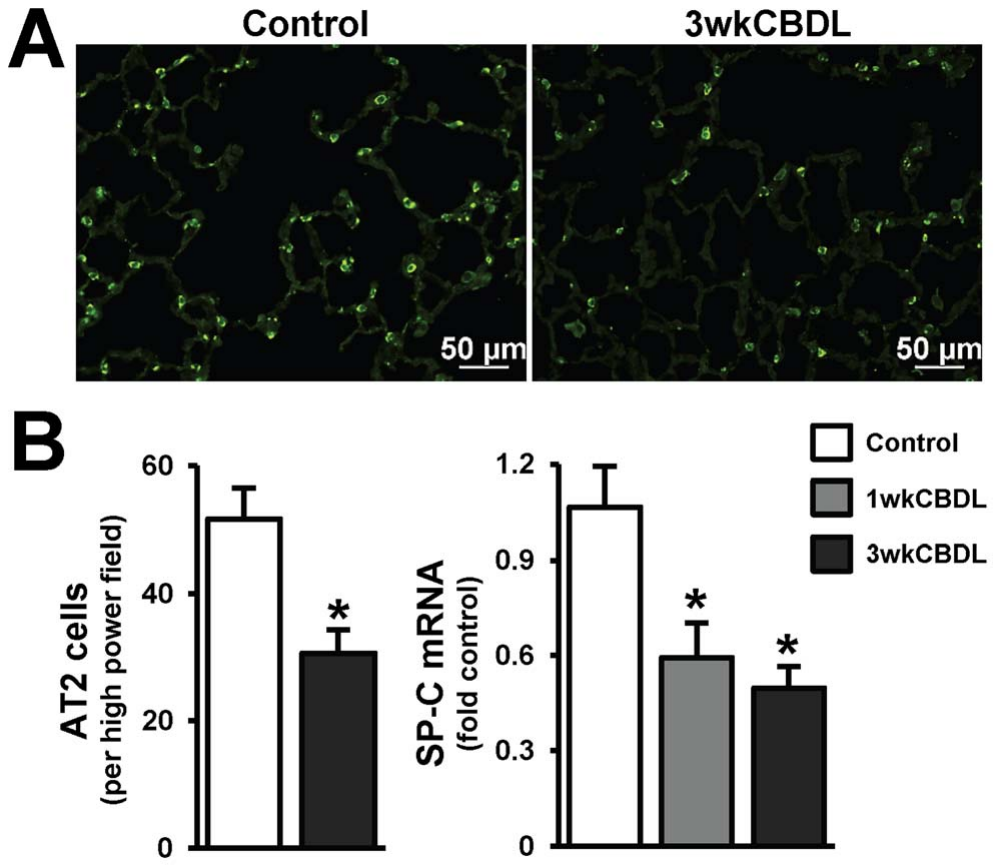
Alveolar type II epithelial cell (AT2) numbers and SP-C mRNA levels after CBDL. (**A**) Representative immunofluorescence images of SP-C staining in alveolar type II epithelial cell (AT2 cells, in green). (**B**) Quantitation of SP-C positive AT2 cells and pulmonary SP-C mRNA levels after CBDL by real-time RT-PCR. Cell counting was performed in a blinded fashion. CBDL resulted in decreased numbers of AT2 cells accompanied by reduced SP-C mRNA levels in lung. Scale bar = 50 µm. Values are expressed as means ± SME. * *P*<0.05 compared with control.

Among the surfactant proteins, SP-C expression is restricted to AT2 cells and it is the most hydrophobic thereby stabilizing the alveolar surface in mammalian lung. As a measure of AT2 cell integrity, we analyzed SP-C mRNA levels and immunohistochemical localization and counted the numbers of AT2 cells (SP-C positive) (Figure 3). Relative to control animals, SP-C mRNA levels began declining within 1 week after CBDL, and progressively decreased over 2 to 3 weeks. Similarly, in control animals, AT2 cells with bright and clear SP-C staining were distributed predominately at the corners of alveoli. In 3 week CBDL animals, the numbers of SP-C positive cells were significantly decreased in the lung supporting a reduction in AT2 cells.

### Pulmonary AT2 Cell Fate after CBDL

**Figure 4:**
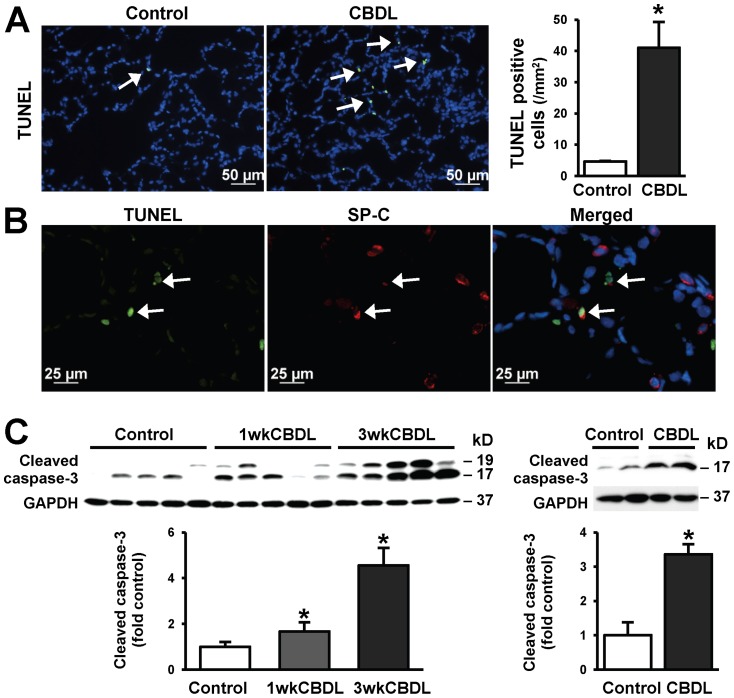
Pulmonary AT2 cell apoptosis after CBDL. (**A**) Representative images of TUNEL staining (green, shown by arrows) and quantitation of TUNEL positive cells in control and 3-week CBDL lung. Scale bar = 50 µm. (**B**) Immunofluorescence double-labeling of SP-C (red) and TUNEL (green) in alveolar regions of 3-week CBDL animal lung. Apoptotic AT2 cells were marked with arrows. Scale bar = 25 µm. (**C**) Representative immunoblots and quantitation of cleaved caspase-3 protein levels in control, 1-week and 3-week lung (left), and in isolated AT2 cells from control and 3-week CBDL lung (right). GAPDH was included as a loading control. Cleaved caspase-3 protein levels were normalized to the levels of GAPDH. Relative to control, CBDL lung had increased apoptotic AT2 cells (TUNEL and SP-C double positive). In parallel, cleaved caspase-3 protein levels were upregulated in both CBDL lung homogenates and isolated AT2 cells. Values are expressed as means ± SME. * *P*<0.05 compared with control.

To define what accounts for the decrease in SP-C synthesis and loss of SP-C positive cells in lung after CBDL, we assessed apoptosis by TUNEL staining and cleaved caspase-3 protein levels (Figure 4A and 4C) and localized apoptotic cells by double-immunoflourescence staining with TUNEL and SP-C (Figure 4B). Relative to control, there was a significant increase in TUNEL positive cells and cleaved caspase-3 protein expression in lung after CBDL. The majority of apoptotic cells co-stained for TUNEL and SP-C indicating that they were AT2 cells. To directly assess AT2 cell apoptosis in response to CBDL, we isolated lung AT2 cells from control and CBDL animals and measured protein levels of cleaved capase-3. The purity of the cells was >90% as determined by SP-C IF staining (Figure S1), consistent with previous studies [40]–[43]. There was a significant increase in cleaved caspase-3 production in AT2 cells isolated from CBDL lung, confirming our *in vivo* observations.

### Modulation of AT2 Cell Surfactant Protein Expression and Apoptosis *In Vitro*

**Figure 5:**
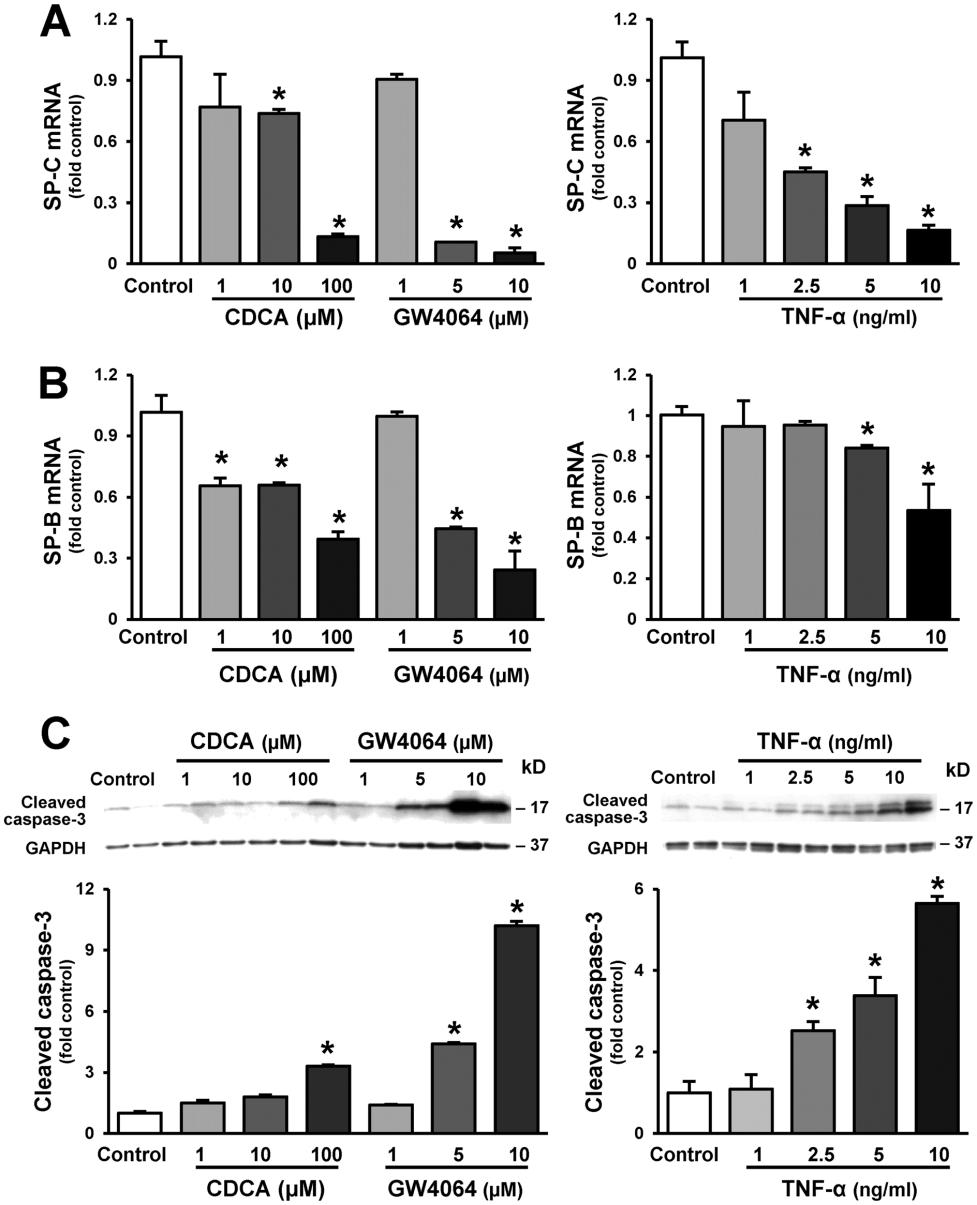
Effects of bile acids and TNF-α on SP-B and SP-C mRNA levels and cleaved caspase-3 expression. Mouse lung epithelial cells (MLE-12) were treated with a varying doses of chenodeoxycholic acid (CDCA, 1–100 µM, 24 hours), GW4064 (a FXR/bile acid nuclear receptor agonist, 1–10 µM, 24 hours) and TNF-α (1–10 ng/ml, 24 hours). The mRNA levels of SP-C and SP-B were measured by real-time RT-PCR. The protein levels of cleaved caspase-3 were assessed by western blots. Three independent experiments were performed. (**A**) Quantitation of SP-C mRNA levels. (**B**) Quantitation of SP-B mRNA levels. (**C**) Representative immunoblots and graphic summaries of cleaved caspase-3 protein levels. GAPDH was included as a loading control. Cleaved caspase-3 protein levels were normalized to the levels of GAPDH. Relative to untreated cells, cells stimulated with CDCA, GW4064 and TNF-α had dose-dependent decreases in SP-B and SP-C mRNA production and a significant increase in cleaved caspase-3 protein expression. Values are expressed as means ± SME. * *P*<0.05 compared with control.

To explore potential mediators and mechanism relative to CBDL that may regulate surfactant protein expression in AT2 cells, we measured circulating and BALF bile acids and TNF-α levels in animal models. Mouse alveolar type II epithelial cells (MLE-12) were treated with bile acids (CDCA), GW4064 (a specific bile acid nuclear receptor FXRα agonist) and TNF-α. The mRNA levels of SPs were measured by real-time RT-PCR. Cell apoptosis was determined by measuring cleaved caspase-3 protein levels. Relative to control, CBDL induced elevated plasma levels of bile acids and TNF-α, while PVL resulted in an increase in bile acid levels alone of much lower magnitude (Table 1). There were no significant differences in the levels of bile acids and TNF-α in BAL fluids between control and CBDL animals (Figure S2). MLE cell line appeared to have abundant SP-B and SP-C mRNAs relative to the levels of SP-A and SP-D mRNAs (Figure S3), consistent with previously reported [39]. Compared to untreated cells, cells treated with CDCA, GW4064 or TNF-α had a significant decrease in both SP-C and SP-B mRNA expression (Figure 5A and 5B), and a significant increase in cleaved caspase-3 protein expression (Figure 5C).

**Table 1:** Circulating bile acids and TNF-α levels in CBDL and PVL animals.

	Control	3 wkCBDL	3 wkPVL
**Bile acids (µmol/L)**	7.28±2.85	109.36±12.10*	48.23±15.45*
**TNF-α (pg/ml)**	1.42±0.54	518.00±160.43*	4.68±3.12

Values are expressed as means ± SME; n = 5–8 animals per group. CBDL, common bile duct ligation; PVL, partial portal vein ligation.

* *P*<0.05 compared with control.

### Pulmonary Function Tests (PFT) in Liver Patients with or without HPS

To determine whether there is evidence for loss of alveolar capacity or functional area in patients with HPS, we performed a retrospective analysis of spirometry parameters obtained from a prospective Pulmonary Vascular Complications of Liver Disease Study (PVCLD) cohort [44] (Table 2). We found that HPS patients, relative to non-HPS patients, had significantly lower values for the forced expiratory volume in the first second (FEV1) and the forced vital capacity (FVC) with preserved FEV1/FVC ratio consistent with restrictive changes.

**Table 2:** Spirometric results in liver patients with and without HPS.

	Non-HPS (n = 146)	HPS (n = 72)	P value
**FEV1 (% predicated)**	90.52 (88.12–92.93)	85.47 (82.22–88.72)	0.015
**FVC (% predicated)**	90.58 (88.15–93.01)	86.33 (82.98–89.67)	0.04
**FEV1/FVC (%)**	78.06 (77.11–79.02)	77.40 (76.19–78.61)	0.41

Values are expressed as percentages and means (95% confidence intervals). FEV1, forced expiratory volume in the first second; FVC, forced vital capacity.

## Discussion

The central feature of clinical HPS is abnormal pulmonary gas exchange driven by the mismatch of ventilation-perfusion or diffusion-perfusion occurring at the alveolar terminal respiratory units [45], [46]. Over the past decade, studies in human and CBDL HPS have focused on dilation and angiogenesis in the pulmonary microvascular compartment which result in altered blood flow and impaired gas diffusion capacity across the alveolar-capillary membrane [11], [24], [47]. However, whether the respiratory epithelial compartment is affected in chronic liver disease has not been well defined. In the present study, we evaluated the functional properties of alveolar epithelial cells and lung morphology after CBDL up to 3 wk to define if the epithelial compartment is affected. We have found a selective decrease in pulmonary surfactant protein levels accompanied by AT2 cell apoptosis and a reduction in alveolar airspace, which occurs as HPS develops after CBDL. These alterations are not seen in PVL animals where HPS does not develop. Moreover, bile acids and TNF-α treatment increased cell apoptosis and decreased surfactant protein expression in cultured AT2 cells. Accordingly, we found a selective increase in restrictive pulmonary function abnormalities in patients with HPS relative to non-HPS cirrhotics consistent with loss of alveolar airspace. These findings support that alterations in alveolar structure and function may occur in both experimental and human HPS.

Pulmonary surfactant is a complex mixture of lipids and proteins that lines the alveolar surface. The protein portion contains four surfactant associated proteins and plays a crucial role in the maintenance of surfactant stability, alveolar integrity and respiratory function as well as in the regulation of lung inflammation and injury [15], [48]. In the present study, we found a reduction in all lung surfactant proteins (SP-A, SP-B, SP-C and SP-D) after CBDL which has not been previously identified. This reduction was accompanied by a decrease in total lung size by water displacement and in alveolar size indicated by a reduction in alveolar mean chord length. These morphologic alterations support that altered surfactant protein production may disrupt alveolar integrity and lead to alveolar collapse. Our findings are consistent with and may explain results from a recent study focusing on lung respiratory mechanics in CBDL animals, which found a reduction in tidal volume, minute ventilation and mean inspiratory flow rate attributable to uneven distribution of alveolar ventilation [49]. These results are unique relative to other primary lung diseases associated with a reduction in surfactant in that lung injury is not a significant feature in HPS. Additional alterations including diaphragmatic and respiratory muscle wasting particularly after prolonged CBDL could also contribute to changes in respiratory mechanics [50]. However, our finding that restrictive PFT changes are also found in HPS cirrhotics but not in non-HPS cirrhotics suggest that alveolar alterations may be unique to HPS.

The mechanisms for the reduced surfactant protein production after CBDL are not fully defined. AT2 cells are the only lung cells that produce all four surfactant proteins and SP-C biosynthesis occurs exclusively in AT2 cells. In the present study, we hypothesized that two potential pathways might lead to decreased SP production in lung; alveolar type 2 epithelial cell apoptosis and decreased surfactant protein expression. Our *in vivo* findings showing increased numbers of TUNEL positive AT2 cells and increased cleaved caspase-3 levels in lung and isolated AT2 cells from CBDL confirm AT2 cell apoptosis. Our *in vitro* findings that bile acids (CDCA), a specific bile acid nuclear receptor FXRα agonist and TNF-α, each induced both cell apoptosis and decreased surfactant protein expression in cultured AT2 cells define potential pathways that drive apoptosis and support that effects on surfactant protein expression may also occur. Elevated bile acid and TNF-α levels in plasma are found in human cirrhosis and in animal models including CBDL and are recognized to induce cell apoptosis through the activation of extrinsic or intrinsic pathways in a number of cell types [23], [29], [51], [52]. Relative to CBDL, PVL lung does not develop alveolar epithelial alterations and TNF-α levels are not increased in this situation and levels of serum bile acids although higher than normal are reported to be significantly lower than 3-week CBDL. Whether the magnitude of bile acid level increase or a synergetic effect of bile acids and TNF-α after CBDL contribute to the selective alterations in AT2 cells after CBDL are not defined. In addition, increased mononuclear cell infiltration in the lung is associated with decreased surfactant protein production in some inflammatory pulmonary diseases [53], [54]. In experimental HPS, monocytes are recruited to the pulmonary intravascular space via altered chemokine/receptor expression (such as fractalkine/CX3CL1) [55]. Whether these cells also produce inflammatory cytokines or mediators that influence AT2 cells needs further investigation.

In normal lung, effective pulmonary arterial gas exchange is achieved by a proper alveolar ventilation (V_A_)/perfusion (Q) ratio. In previous studies, we and other have clearly defined that intrapulmonary vasodilatation causes abnormalities in capillary blood flow that lead to V/Q mismatch and drives impaired oxygenation in human and experimental HPS [10], [24], [52], [56]. Our observations in the present study extend these findings and support that a ventilation defect due to loss of alveolar type II cell surfactant may be also present in experimental HPS and may augment V/Q mismatch. Although chronic liver disease has been associated with restrictive ventilatory defects in certain situations [3], [4], [12], the unique combination of perfusion impairments and ventilatory defects in experimental HPS may explain the potential for significant gas exchange abnormalities in the absence of marked histologic abnormalities. The findings of restrictive defects in human HPS patients support that similar events may occur in human disease but requires further evaluation.

**Figure 6:**
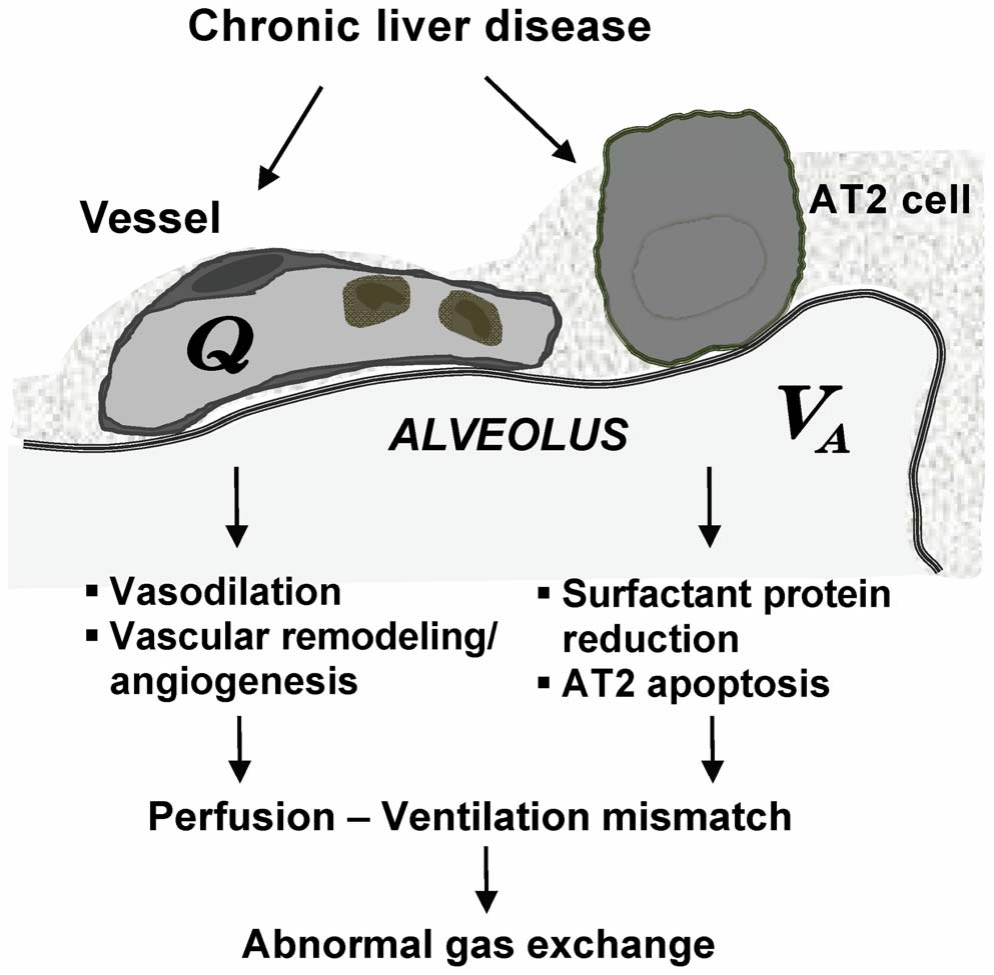
Current understanding of cellular mechanisms in the development of experimental hepatopulmonary syndrome. V_A_, alveolar ventilation; Q, alveolar blood perfusion.

In the present study, we have identified alterations in alveolar structure and function as a novel potential contributor to pulmonary gas exchange abnormalities in experimental HPS. These alterations are associated with specific effects on AT2 cells that result in a decline in surfactant expression. These results extend our understanding of the mechanisms of ventilation-perfusion abnormalities in this disorder and may explain prior findings in CBDL animals (Figure 6). Moreover, they provide a potential novel focus for pathophysiologic investigation and therapeutic intervention.

## Supporting Information

Figure S1 **SP-C immunofluorescence staining of isolated AT2 cells from animal lung.** A SP-C negative cell was shown (white arrow). The purity of the cells was >90% as determined by SP-C IF staining.
(TIFF)

Figure S2 **Bile acids and TNF-α levels in BALFs from control and CBDL animals.** BAL fluids were obtained from control and 3-week CBDL animals, the concentrations of total bile acids and TNF-α were measured using commercial available kits. There were no significant differences in both bile acids and TNF-α levels between control and CBDL groups. Values are expressed as means ± SME.
(TIFF)

Figure S3 **Amplification plot showing differential expression levels of SP mRNAs in MLE-12 cells.** The basal mRNA levels of four SPs in MLE-12 cell line were assessed by real-time RT-PCR. 18S rRNA was included as an endogenous control. Relative to SP-A and SP-D, SP-B and SP-C mRNAs were expressed abundantly in MLE-12 cell line.
(TIFF)
