# Supplementary figures and images for: Alveolar Type II Epithelial Cell Dysfunction in Rat Experimental Hepatopulmonary Syndrome (HPS)
Source: PLoS One. 2014 Nov 24;9(11):e113451. doi: 10.1371/journal.pone.0113451 (PMC4242631; doi:10.1371/journal.pone.0113451)

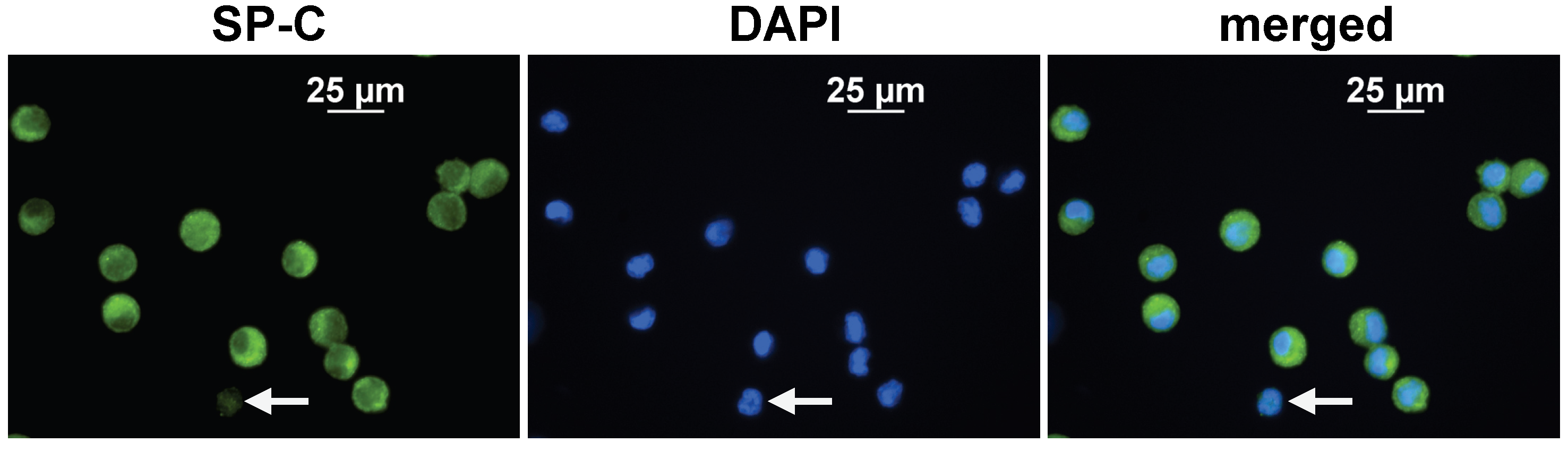

Supplement: Figure S1 — SP-C immunofluorescence staining of isolated AT2 cells from animal lung. A SP-C negative cell was shown (white arrow). The purity of the cells was >90% as determined by SP-C IF staining. (TIFF) [file pone.0113451.s001.tiff]

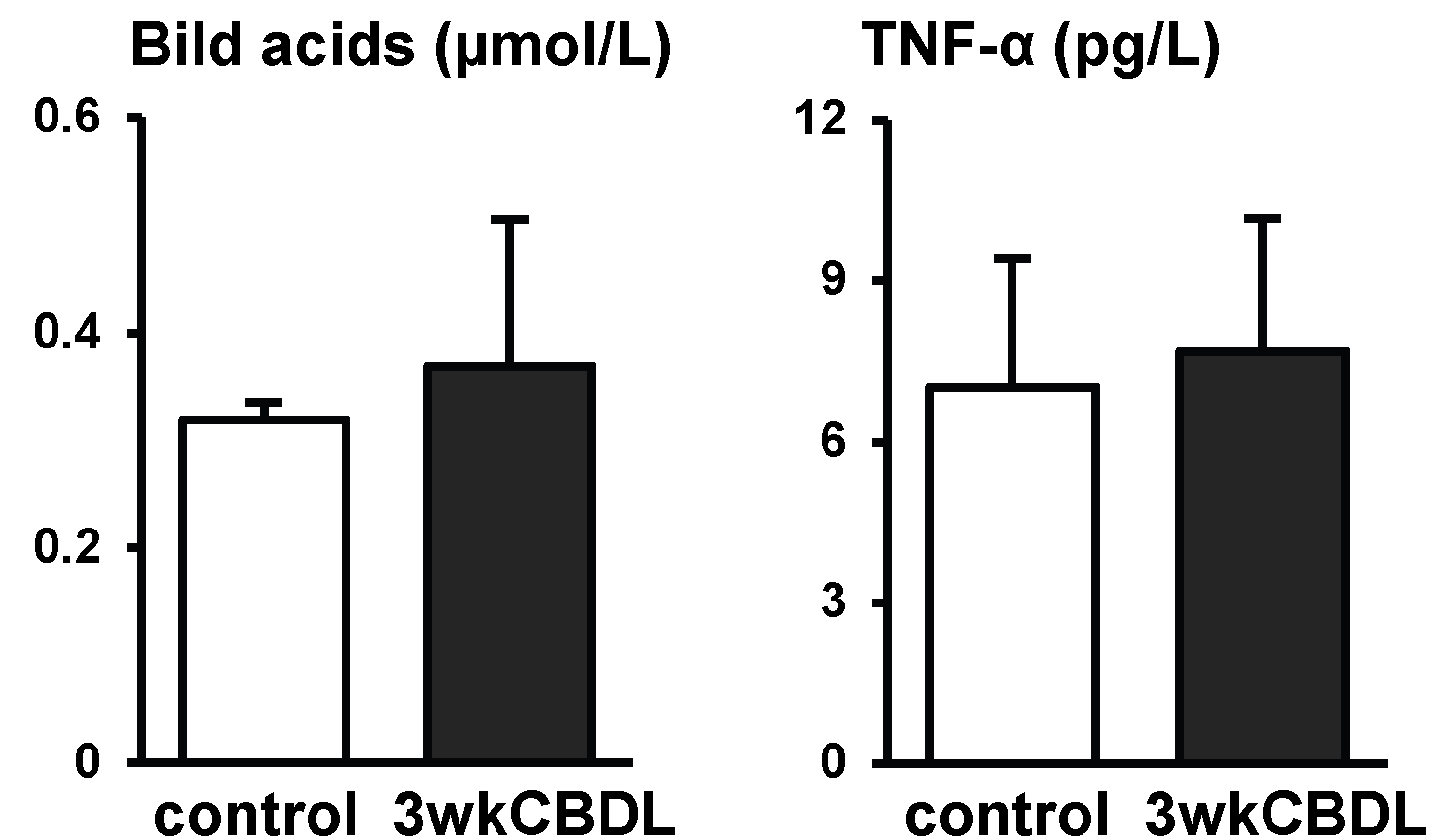

Supplement: Figure S2 — Bile acids and TNF-α levels in BALFs from control and CBDL animals. BAL fluids were obtained from control and 3-week CBDL animals, the concentrations of total bile acids and TNF-α were measured using commercial available kits. There were no significant differences in both bile acids and TNF-α levels between control and CBDL groups. Values are expressed as means ± SME. (TIFF) [file pone.0113451.s002.tiff]

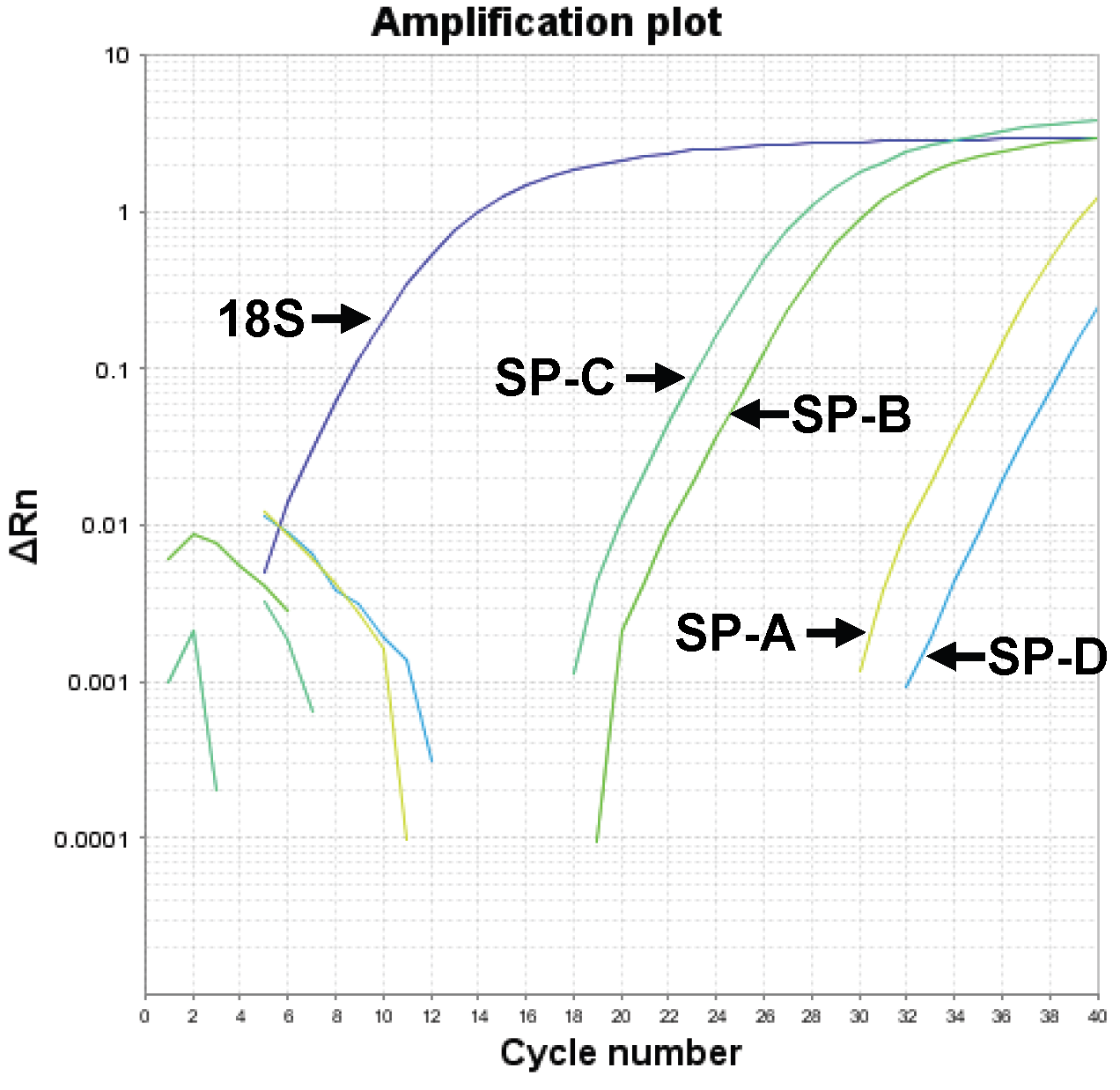

Supplement: Figure S3 — Amplification plot showing differential expression levels of SP mRNAs in MLE-12 cells. The basal mRNA levels of four SPs in MLE-12 cell line were assessed by real-time RT-PCR. 18S rRNA was included as an endogenous control. Relative to SP-A and SP-D, SP-B and SP-C mRNAs were expressed abundantly in MLE-12 cell line. (TIFF) [file pone.0113451.s003.tiff]
